# 

**Published:** 1896-07

**Authors:** 


### CONTENTS.

COMMUNICATIONS.

The Value of Differential Diagnosis in Dentistry...... 313

PROCEEDINGS.

The Mississippi Dental Association...... 322
Monthly Digest...... 328

COMMENCEMENTS.

Ohio Medical University—Dental Department...... 345
Chicago College of Dental Surgery...... 345
American College of Dental Surgery...... 346
Western Dental College of Kansas City...... 347
Royal College of Dental Surgeons of Ontario...... 348
Ohio College of Dental Surgery...... 349
Columbian University—Dental Department...... 349
Atlanta Dental College...... 350
Cincinnati College of Dental Surgery...... 350
New York College of Dentistry...... 351
Harvard Dental College...... 352
University of Tennessee—Dental Department...... 352
University of California—College of Dentistry...... 353
University of Michigan - College of Dental Surgery...... 353
University College of Medicine—Dental Department...... 354
University of Denver—Dental Department...... 355
Boston Dental College...... 355
Louisville College of Dentistry...... 356
National University—Dental Department...... 356
University of Pennsylvania -Department of Dentistry...... 357
Western Reserve University—Dental Department...... 357

SELECTIONS.

Odor as a Symptom of Disease...... 358

NOTICES.

American Dental Association...... 360, 361
National Association of Dental Faculties...... 360
The National Association of Dental Examiners...... 361
Twelfth International Medical Congress...... 361

EDITORIAL.

Annual Meeting of the Indiana State Dental Society...... 362
The Southern Dental Association's Proceedings...... 363
Book Notice...... 363
Obituary...... 364

